# The Association of Depressive Symptoms with Inflammatory Factors and Adipokines in Middle-Aged and Older Chinese

**DOI:** 10.1371/journal.pone.0001392

**Published:** 2008-01-02

**Authors:** An Pan, Xingwang Ye, Oscar H. Franco, Huaixing Li, Zhijie Yu, Jing Wang, Qibin Qi, Wenjia Gu, Xinghuo Pang, Hong Liu, Xu Lin

**Affiliations:** 1 Key Laboratory of Systems Biology, Institute for Nutritional Sciences, Shanghai Institutes for Biological Sciences, Chinese Academy of Sciences and Graduate School of the Chinese Academy of Sciences, Shanghai, China; 2 Unilever Corporate Research, Colworth House, Sharnbrook, Bedfordshire, United Kingdom; 3 Beijing Center for Diseases Control and Prevention, Beijing, China; 4 Shanghai Center for Diseases Control and Prevention, Shanghai, China; Cornell University, United States of America

## Abstract

**Background:**

Studies in Western populations find that depression is associated with inflammation and obesity. The present study aimed to evaluate the relation of depressive symptoms with inflammatory factors and adipose-derived adipokines in middle-aged and older Chinese.

**Methodology/Principal Findings:**

Data were from 3289 community residents aged 50–70 from Beijing and Shanghai who participated in the Nutrition and Health of Aging Population in China project. Depressive symptoms were defined as a Center for Epidemiological Studies of Depression Scale (CES-D) score of 16 or higher. Plasma concentrations of C-reactive protein (CRP), interleukin-6 (IL-6), adiponectin, resistin, plasminogen activator inhibitor-1 (PAI-1) and retinol binding protein 4 (RBP4) were measured. Of the 3289 participants, 312 (9.5%) suffered from current depressive symptoms. IL-6 level was higher in participants with depressive symptoms compared to their counterparts in the crude analyses (1.17 vs. 1.05 pg/mL, *p* = 0.023) and this association lost statistical significance after multiple adjustments (1.13 vs. 1.10 pg/mL, *p* = 0.520). Depressive symptoms were not associated with increased mean levels of any other inflammatory factors or adipokines in the unadjusted or adjusted analyses.

**Conclusions/Significance:**

We found no evidence that depressive symptoms were associated with inflammatory factors and adipokines in the middle-aged and older Chinese populations. Prospective studies and studies in clinically diagnosed patients are needed to confirm our results and clarify the relation of depression with inflammatory factors and adipokines.

## Introduction

Depression is a mental disorder significantly associated with increased risk of the onset and mortality of cardiovascular diseases (CVD) [1], [2]. Several mechanisms have been proposed to explain these observed associations, including poor compliance to medications [3], elevated platelet reactivity [4], reduced heart rate variability [5], increased cortisol and catecholamine levels [6], [7]. Of more recent interest is the role of inflammation and obesity in the development of CVD [8], [9] and their potential relation to depression [10], [11].

To date, numerous studies have suggested that chronic inflammation is an intrinsic part of atherosclerosis [12] and plays an important role in the pathogenesis of CVD [8]. It has become established that C-reactive protein (CRP), a marker of systemic inflammation, is an independent predictor of CVD [13], [14]. Interleukin-6 (IL-6), an inflammatory cytokine and major inducer of the secretion of CRP, is also associated with CVD [15]. Results about the correlation between depression and inflammation are inconsistent. Most studies found positive association [16]–[25], and some reported null association [26], [27], whereas one study even found inverse association [28]. Nevertheless, all of the above studies were conducted in Western countries and none has investigated this association in Chinese populations.

Adipose tissue is now regarded as an endocrine organ and secretes several adipokines, such as adiponectin, resistin, plasminogen activating inhibitor-1 (PAI-1) and retinol binding protein 4 (RBP4) [29]–[31]. In obesity, increased production of most adipokines affects multiple functions such as appetite and energy balance, lipid metabolism, insulin sensitivity, immunity, blood pressure, angiogenesis, and hemostasis, all of which are linked with CVD [32]. The association between obesity and depression has been intensively studied in western populations and a recent comprehensive review suggests that they are positively correlated, particularly in women [33]. While a large cross-sectional study in Chinese elderly in Hong Kong found negative association [34] and few studies were done in mainland China. Meanwhile, the association between depression and various adipose-derived adipokines is sparsely studied and the few existing findings are controversial [35]–[39].

Therefore, we sought to evaluate the association of clinical relevant depressive symptoms with several inflammatory markers and adipokines in a cross-sectional study of 3289 middle-aged and older Chinese.

## Materials and Methods

### Participants

The present study is part of the Nutrition and Health of Aging Population in China project and the detailed study design has been described previously [40], [41]. In brief, a stratified, multi-stage sampling method was adopted. One rural county and two urban districts representing low, middle and upper socioeconomic levels were chosen in both Beijing and Shanghai. In each selected county or district, villages or communities were randomly selected and all eligible potential participants were listed and asked the intention of inclusion. The sampling procedure ended when we fulfilled our designed sample size of 3200. Only one person per household was randomly selected and a total of 3289 eligible participants (1458 men and 1831 women) were finally recruited. To be eligible, participants should be non-institutionalized individuals of 50–70 years old who were stable residents for at least 20 years in Beijing or Shanghai. Participants were excluded from the study if they had one of the following conditions: self-care disabilities; severe psychological disorders; newly diagnosed with cancer, coronary heart disease, stroke, Alzheimer's disease and dementia within the 6 month period before the start of the study; or currently diagnosed with tuberculosis, AIDS and other communicable diseases. The study was approved by the Institutional Review Board of the Institute for Nutritional Sciences, and signed informed consent was obtained from each participant.

### Depressive Symptoms

During the home interview, clinically relevant depressive symptoms were measured with the 20-item Center for Epidemiologic Studies Depression Scale (CES-D) [42], which has been previously validated in Chinese populations [43], [44]. To identify respondents with a level of depressive symptoms that is clinically relevant, we used the generally accepted cutoff point of 16, which has a good validity for major depression [45].

### Inflammatory Markers

Peripheral venous Ethylene Diamine Tetraacetic Acid (EDTA) blood samples were collected in the morning after at least 8 hours' fasting. The blood samples were centrifuged at 4°C, 3,000 rpm for 15 minutes. After being frozen, the samples were shipped in dry ice to the Institute for Nutritional Sciences and stored at −80°C until analysis. CRP was measured by a particle-enhanced immunoturbidimetric assay (Orion Diagnostica, Espoo, Finland) using microparticles coated with anti-human CRP antibodies. The lower detection limit of the assay was 0.25 mg/L and the detailed assay procedure was described previously [40]. IL-6 was measured with high-sensitivity enzyme-linked immuno-sorbent assay (Quantikine HS IL-6 Immunoassay, R&D Systems Inc., Minneapolis, MN). The linear range was 0–10 pg/mL with low detect limit of 0.04 pg/mL. All undetectable CRP values (<0.25 mg/L) were replaced with 0.12 mg/L and IL-6 level lower than 0.04 pg/mL was set to 0.02 pg/mL. A total of 96 individuals were excluded from IL-6 analysis due to gross hemolysis or lipemia according to the kit instructions.

### Adipokines

Plasma adiponectin, resistin and active PAI-1 concentrations were measured by Luminex xMAP™ Technology (Linco Research Inc, St Charles, Mo) on a Bio-Rad Multiplex Suspension Array System. The sensitivity of the assay was 145.5, 6.7 and 1.3 pg/mL for adiponectin, resistin and active PAI-1, respectively, with an intra-assay coefficient of variation (CV) of 1.4–7.9% and an inter-assay CV <21%. Adipokines measurements were done simultaneously with IL-6 measurements, therefore the same 96 samples were excluded from analyses.

Plasma RBP4 was measured in duplicate by an in-house developed and validated sandwich ELISA, with an intra-assay CV of 1.8–7.6% and an inter-assay CV of 3.7–8.8%. Detailed assay procedure has been described elsewhere [46].

### Other Biomarkers

Total cholesterol, triglycerides, and glucose were measured enzymatically on an automatic analyzer (Hitachi 7080, Japan) with reagents purchased from Wako Pure Chemical Industries (Osaka, Japan). Fasting insulin was determined by radioimmunoassay (Linco Research, MO).

### Covariates

Covariates included characteristics associated with depressive symptoms, inflammation and adipokines as suggested by previous publications. The study team was trained to conduct the face-to-face home interviews using a standard questionnaire. Socio-demographic variables included age, gender, geographic location (Beijing/Shanghai), residential region (urban/rural) and educational level (≤9 or >9 years in school). Current smoking status (yes/no) and alcohol drinking status (yes/no) were also assessed. The physical activity level for each individual was classified as low, moderate, and high according to the International Physical Activity Questionnaire (short last 7 day format) scoring protocol [47] with minor modification. Body height and weight of the participants were measured and body mass index (BMI) was then calculated as weight (kg)/height^2^ (m^2^). Participants were categorized as non-obese (<28.0 kg/m^2^), obese (≥28.0 kg/m^2^) according to the criteria for Chinese [48]. Blood pressure was measured by using an electronic blood pressure monitor (Omron HEM-705CP, OMRON Healthcare INC., Illinois). Information of anti-inflammatory medications in the preceding two weeks and presence of comorbidities (diabetes mellitus, cardiovascular diseases, cerebrovascular diseases, cancer, asthma, chronic bronchitis, emphysema, pulmonary heart disease, gastrointestinal ulcer, arthritis, rheumatic and rheumatoid arthritis) were obtained. Diabetes mellitus was defined as fasting glucose ≥7.0 mmol/L, or previously diagnosed type 2 diabetes or on treatment with oral anti-diabetic agents or insulin. Other comorbidities were self-reported. Binary categories of respondents were created according to the presence or absence of any of the above comorbidities.

### Statistical Analyses

Data were analyzed using Stata 9.2 (StataCorp, College Station, Texas). A two-sided *p* value of 0.05 or less was defined as statistically significant. Differences in proportions and means of covariates between participants with and without depressive symptoms were assessed using χ^2^ and *t* test statistics, respectively. General linear regression analyses were used to compare mean levels of inflammatory markers and adipokines between participants with and without depressive symptoms, adjusted for potential confounding variables (geographic location, residential region, age, gender, educational level, smoking and drinking status, physical activity level, BMI, log-triglyceride, log-insulin, log-total cholesterol, use of anti-inflammatory medications and presence of comorbidity, listed in table 1). For these analyses, natural log-transformations were performed to approximate normality of CRP, IL-6, adiponectin, resistin, PAI-1 and RBP4, as they were not normally distributed.

**Table 1:** Characteristics of Study Participants According to Depressive Symptoms

	n	Depressive Symptoms (n = 312)	No Depressive Symptoms (n = 2977)	*p* Values^a^
Age (years)	3289	58.26±6.17	58.64±5.99	0.285
Women	1831	214 (68.6%)	1617 (54.3%)	<0.001
Geographic Location				<0.001
Beijing	1641	224 (78.2%)	1397 (46.9%)	
Shanghai	1648	68 (21.8%)	1580 (50.1%)	
Residential Region				0.135
Rural	1649	143 (45.8%)	1697 (50.3%)	
Urban	1640	169 (54.2%)	1480 (49.7%)	
Current Smoker	920	77 (24.7%)	843 (28.3%)	0.173
Current Alcohol Drinker	939	82 (26.3%)	857 (28.8%)	0.351
Physical Activity Level				<0.001
Low	245	46 (14.7%)	199 (6.7%)	
Moderate	1381	121 (38.8%)	1260 (42.3%)	
High	1663	145 (46.5%)	1518 (51.0%)	
Education Level (>9 years in school)	757	52 (16.7%)	705 (23.7%)	0.005
Anti-inflammatory Drugs	471	85 (27.2%)	386 (13.0%)	<0.001
Presence of Comorbidity	1295	152 (48.7%)	1143 (38.4%)	<0.001
Presence of Obesity^b^	487	54 (17.3%)	433 (14.5%)	0.191
Body Mass Index (kg/m^2^)^c^	3289	24.85±3.90	24.42±3.55	0.064
Triglyceride (mmol/L)^c^	3289	1.39±0.97	1.39±1.08	0.523
Total Cholesterol (mmol/L)^c^	3289	4.80±0.99	4.69±0.98	0.054
Fasting Plasma Insulin (pmol/L)^c^	3285	16.53±9.28	15.32±9.49	0.008
Systolic blood pressure (mmHg)^c^	3289	142.8±22.2	139.8±22.5	0.028

Data are presented as n (%) or mean±SD.

a *p* values are based on χ^2^ tests for categorical variables and Student's *t*-tests for continuous variables.

b Obesity is defined as having a body mass index ≥28 kg/m^2^.

c Data are not normally distributed, *p* values were calculated based on log-transformed data.

To further examine the associations, we used logistical regression with elevated inflammatory factors or adipokines as a dichotomous outcome variable and current depressive symptoms as a predictor variable. Elevated inflammatory factors or adipokines were defined as having values in the top quartile of each biomarker.

## Results

Of the 3289 participants, 312 (9.5%) suffered from current depressive symptoms as indicated by a CES-D score ≥16. The prevalence rates of depressive symptoms were higher in women and participants from Beijing than their counterparts (Table 1). The substantial disparity between participants from Beijing and Shanghai may be partly due to large variations in culture, lifestyle, dietary habits, social support, living condition, and climate between the two cities [41]. Presence of depressive symptoms was significantly associated with low physical activity level and low educational level. Higher prevalence of depressive symptoms was found in participants using anti-inflammatory drugs and with comorbidities (particularly in participants with self-reported CVD, data not shown). Participants with depressive symptoms had significantly higher fasting insulin concentrations (14.51 vs. 13.33 µU/mL, *p* = 0.008), higher systolic blood pressure (142.8 vs. 139.8 mm Hg, *p* = 0.028) compared to those without depressive symptoms. People with depressive symptoms had higher BMI (24.85 vs. 24.42 kg/m^2^, *p* = 0.064) and higher prevalence of obesity (17.3% vs. 14.5%, *p* = 0.191) than their counterparts, while it did not reach statistical significance.

Current depressive symptoms were only associated with increased IL-6 level in the crude analyses and this association lost statistical significance after multiple adjustment (Table 2). Depressive symptoms were not associated with increased mean levels of any other inflammatory factors or adipokines in unadjusted or adjusted analyses. When CES-D score was entered as a continuous variable, we observed no evidence for a linear or non-linear association of depressive symptoms with increased levels of any of the factors evaluated (data not shown).

**Table 2:** Plasma Levels of Inflammatory Factors and Adiopokines in Study Participants According to Depressive Symptoms

	Unadjusted Geometric Mean (95% CI)			Adjusted Geometric Mean (95% CI)^a^		
	Depressive Symptoms	No Depressive Symptoms	*p* Value^b^	Depressive Symptoms	No Depressive Symptoms	*p* Value^b^
CRP (mg/L)	0.79 (0.69–0.91)	0.70 (0.67–0.73)	0.107	0.78 (0.68–0.90)	0.80 (0.74–0.86)	0.803
IL-6 (pg/mL)	1.17 (1.06–1.29)	1.05 (1.02–1.08)	0.023	1.13 (1.03–1.25)	1.10 (1.04–1.16)	0.520
Adiponectin (µg/mL)	13.06 (11.93–14.30)	12.77 (12.42–13.13)	0.622	11.80 (10.82–12.86)	12.07 (11.48–12.68)	0.585
Resistin (ng/mL)	9.64 (8..86–10.50)	8.97 (8.76–9.19)	0.110	9.59 (8.81–10.44)	9.23 (8.79–9.70)	0.362
PAI-1 (ng/mL)	9.06 (7.87–10.43)	8.80 (8.39–9.24)	0.713	9.07 (7.85–10.49)	10.32 (9.50–11.21)	0.068
RBP4 (µg/mL)	38.21 (36.86–39.61)	38.39 (37.98–38.81)	0.791	38.94 (37.60–40.33)	39.85 (39.06–40.65)	0.173

Data are presented as Geometric Mean (95% CI).

CRP, C-reactive protein; CI, confidence interval; IL-6, interleukin-6; PAI-1, plasminogen activator inhibitor-1; RBP4, retinol binding protein 4.

a Adjusted for geographic location, residential area, age, sex, body mass index, smoking status, drinking status, physical activity level, educational level, comorbidity status, log-insulin, log-triglyceride, log-total cholesterol, log-systolic blood pressure, and use of anti-inflammatory drugs.

b *p* value was calculated based on log-transformed data.

We confirmed in logistical regression analyses that depressive symptoms were not associated with elevated levels of any of these biomarkers (Table 3). No associations were either found in subgroups stratified by geographic location, gender, obesity or anti-inflammatory medications usage (data not shown).

**Table 3:** Adjusted Odd Ratio of Elevated Inflammatory Factors and Adiopokines Associated with Depressive Symptoms in the Study Participants^a^

	Risk of Elevated Biomarkers b (Depressive Symptoms: Yes vs. No)	*p* Value
CRP (≥1.51 mg/L)	0.99 (0.74–1.31)	0.920
IL-6 (≥1.60 pg/mL)	1.01 (0.77–1.34)	0.931
Adiponectin (≥21.72 µg/mL)	1.02 (0.76–1.37)	0.909
Resistin (≥14.01 ng/mL)	1.14 (0.86–1.49)	0.363
PAI-1 (≥19.45 ng/mL)	0.79 (0.58–1.08)	0.138
RBP4 (≥46.85 µg/mL)	0.95 (0.71–1.27)	0.714

Data are presented as odd ratio (95% CI).

CRP, C-reactive protein; IL-6, interleukin-6; PAI-1, plasminogen activator inhibitor-1; RBP4, retinol binding protein 4.

a Adjusted for geographic location, residential area, age, sex, body mass index, smoking status, drinking status, physical activity level, educational level, comorbidity status, log-insulin, log-triglyceride, log-total cholesterol, log-systolic blood pressure, and use of anti-inflammatory drugs.

b Elevated inflammatory factors or adipokines were defined as having values in the top quartile of each biomarker.

## Discussion

In a sample of 3289 community residents from Beijing and Shanghai, we found no evidence that depressive symptoms were associated with increased levels of various inflammatory factors (CRP and IL-6) and adipokines (adiponectin, resistin, PAI-1 and RBP4). To the best of our knowledge, the present study is the first to simultaneously investigate the association of depressive symptoms with inflammatory factors and adipokines in Chinese populations.

### Depressive Symptoms and Inflammatory Factors

The relation between depression and inflammation has been widely studied with inconsistent results reported. Two studies found null relation [26], [27] and one study reported inverse association [28]. While some other large epidemiological studies found positive relations in older American persons [16], [17], young American adults [18], [19] and adult Europeans [20], [21], even after adjustment for various confounding factors. Nevertheless, some other studies suggested that this association might be mediated by various confounders (such as poor health status, obesity and lifestyle). For example, studies in adults aged ≥60 years living in Holland [22], American persons free of CVD aged >65 years [23], young Finnish adults [24] and community-dwelling men aged ≥70 years [25]. In line of the above studies [22]–[25], we found in the present study that depressive symptoms were associated with increased level of IL-6 in the crude analyses, while this relation became non-significant after adjustment for various confounding factors (including demographic and lifestyle information, comorbidity, use of anti-inflammation medications and other related biomarkers). Therefore, further studies are still needed to evaluate whether inflammation and depression are correlated in Chinese populations and the nature of this association.

### Depressive Symptoms and Adipokines

Previous studies have found that depression is positively associated with obesity [33], while a large cross-sectional study in elderly Chinese in Hong Kong found negative association [34]. Our study is the first to investigate this association in mainland China and we found that they were not correlated. One potential explanation for our observed null association may be due to the inter-influence and osmosis between Chinese traditional culture and the modern opinion on obesity. Parallel to the “jolly fat” hypothesis [49] and a well-known idiom of “happy mind and fat body” in Chinese culture [34], being fat is a symbol of wealth and power rather than unhappiness in traditional Chinese culture. However, in modern China where there is increasing prevalence of obesity and awareness of associated morbidity and mortality, stigma of obesity and lower self-esteem of overweight people may be caused. Therefore, it is really difficult to determine the relation between depression and obesity, as we have no information of the participants' attitudes towards obesity.

The association between depression and adipose-derived adipokines is a sparsely studied area and the direction remains unclear. Leo et al. [35] found in a case-control study that patients with clinical diagnosed major depression displayed lower adiponectin levels compared to the healthy controls and adiponectin significantly correlated with depression severity. While Mamalakis et al. [36] found null association between depression and adiponectin levels in 90 healthy adolescents. Another two case-control studies found that depression was associated with higher levels of PAI-1 activity in men [37] and premenopausal women [38]. Recently, a large longitudinal study (Study of Women's Health Across the Nation, SWAN) [39] found that higher depressive symptoms were related to higher PAI-1 levels in perimenopausal women, while this association disappeared after controlling for various confounding factors, particularly BMI. Our study is the first to simultaneously evaluate the association between depressive symptoms and various adipokines (adiponectin, resistin, PAI-1 and RBP4) in general populations. One reason for the lack of associations may be due to the fact that obesity was not associated with depressive symptoms in our study populations. Meanwhile, the endocrine function of adipose tissue is influenced by both body composition (total fat mass and lean body mass) and fat distribution (visceral and subcutaneous adiposity) [29], which are more accurate parameters of describing obesity status than just BMI alone, further studies are needed to evaluate whether body composition and fat distribution are related to depression. Another reason is that some of these adipokines are not exclusively secreted by adipose tissues and could be secreted by other organs (for example, resistin could be produced in peripheral blood monocytes [50], PAI-1 and RBP4 could be synthesized in the liver, [29], [51]).

### Strengths and Limitations

The main strength of our study is that we used data from a large population-based sample of both genders and from both northern and southern China, which is representative of populations of this age. Furthermore, we carefully controlled for various covariates known to be related to inflammation, adipokines and depression in the analyses.

Admittedly, certain limitations should be considered in interpreting our results. Firstly, the validity of the findings based on the self-reported measure of depression (CES-D) is inferior to the psychiatric diagnostic interview, which is the gold standard. However, the sensitivity of the CES-D to detect major depression is high [41] and has been previously validated in Chinese populations [39], [40]. Secondly, this study was performed in a population with narrow age range (50–70 years old) and may not be generalizable to other age groups. Finally, we are unable to determine the causal direction of any relation (or lack of relation) due to the cross-sectional study design.

### Conclusions

In conclusion, depressive symptoms are not associated with inflammatory factors (CRP and IL-6) and adipokines (adiponectin, resistin, PAI-1, RBP4) in the middle-aged and older Chinese populations. Therefore, the relationship between depression and CVD is unlikely to be explained through direct effects on inflammatory factors and adipokines. Further studies, particularly prospective studies and studies in clinically diagnosed patients, are needed to confirm the results. Studies in different populations and age groups are also warranted.
